# Identifying Core Issues for Basin Management: The Issue Generating Assessment (IGA) Methodology

**DOI:** 10.1007/s00267-024-01981-7

**Published:** 2024-05-14

**Authors:** Yael Salame-Rubin, Eran Feitelson, Richard Laster, Shai Gilad, Ahlam Swetat

**Affiliations:** 1 The Hebrew University of Jerusalem, Jerusalem, Israel; 2 Laster & Gouldman Law Office, Jerusalem, Israel; 3 Independent Scholar, Gaaton, Israel

**Keywords:** Mediterranean streams, Stakeholders, Issue generating assessment (IGA), Integrated basin management

## Abstract

Effective stakeholder engagement is essential for basin management, requiring structured approaches to foster collaboration and consensus. This paper applies the Issue Generating Assessment (IGA) method, which identifies core issues for stakeholder discussion, to basin management. Focusing on the Israeli part of the Hadera Basin, we identify the core issues that should be discussed by stakeholders using the IGA method. To this end 39 participants across 14 sectors evaluating three generic basin management strategies were asked to qualitatively explain their evaluations. By analyzing these explanations utilizing the IGA method, four core issues emerged: (1) Managing uncertainty: addressing climate change and land use impacts on stream flow; (2) Mutual impacts management: handling interactions between the stream and its surroundings; (3) Integration of uses: balancing various stream utilization priorities; (4) Defining natural system functions: determining the role of natural systems. For each core issue, we proposed questions to guide stakeholder discussions. The IGA method is thus found to be useful, and has the potential to foster meaningful dialogue in structured stakeholder meetings, thereby focusing discussions and allowing understandings among stakeholders to be reached as a basis for basin management plans. Such early understandings may contribute to the development of strategies for sustainable basin management.

## Introduction

Integrated basin management has been advocated in order to enhance the provision of ecosystem services, including sufficient clean water for the wellbeing of people within the basin, and allowing for economic growth and social equity (Heathcote 2009; Julio et al. 2021; Watson 2004). Integrated basin management calls for the establishment of appropriate institutions and statutes (Jaspers 2003; Hooper 2005), such as called for in the European Water Framework Directive (Giadoumis and Voulvoulis 2018). It is widely stated that such institutions should facilitate stakeholder involvement for a variety of reasons, not least the information they can bring to the table (Jongman and Padovani 2006; Wehn et al. 2015; Wright and Fritsch 2011). To this end workshops, roundtables and outreach efforts are often conducted (Pargament et al. 2010). Yet, as a recent review shows, there is very little research on how effective stakeholder engagement should be carried out (Lim et al. 2022).

Though largely uncoupled, the calls and tools for stakeholder involvement in basin management are similar to the calls for communicative planning (Watson and de Loe 2021). Practices have been advanced to this end in the planning field since the early 1990s (Healey 1992; Innes 1996). Essentially, communicative planning seeks to involve stakeholders through discursive means in an effort to reach agreements that can serve as a basis for plans. In this sense it is similar to the practices advocated in the basin management field (Murray 2007). However, communicative planning has been critiqued as an approach that does not overcome power differentials (Allemdendiger and Tewdwr-Jones 2002) as its outcomes are largely a function of the identity of those involved in the process, who may not fully represent all the relevant stakeholders (Bicerstaff and Walker 2005). These critiques are relevant also for basin management practices, resulting in limited effects of stakeholders on basin management (Blackstock et al. 2014; van der Heijden and ten Heuvelhof 2012; Watson and de Loe 2021).

As Dery (1984) argued, the way problems are defined sets the agenda. Similarly in the operation research field qualitative problem structuring methods were developed in order to facilitate negotiations, and as a learning method taking actors through a process that hopefully leads to politically feasible solutions (Smith and Shaw 2019). In essence, the issue with respect to basin management thus amounts to finding a way to structure discussions in a way that would focus deliberations in a meaningful and productive way, thereby assisting stakeholders to reach agreements over policies. This is the challenge we address in this paper in the basin management context. To this end we utilize the Issue Generating Assessment (IGA), as advanced by Feitelson (2011), to identify core issues that need to be addressed by stakeholders in basin management.

Integrated river basin management was largely initially advanced in humid settings. In such settings rivers are perennial, serving many purposes, and often are the backbone of human settlements (Dombrowsky et al. 2010). In contrast, basins in Mediterranean settings are typified by high inter annual and inter seasonal precipitation and flow patterns (Bonanda and Resh 2013; Cid et al. 2017). This variance is likely to increase with climate change (Pörtner et al. 2022). Consequently, the periods in which flows cease are likely to increase, as are extreme flooding events (Tzoraki and Nikolaidis 2007). Despite the variance among Mediterranean ecosystems, these basins have many common features as all species adapt to the high variance in precipitation, flow, and temperatures (Bonanda and Resh 2013). In this study we analyze a Mediterranean basin as our case study. Hence the results of the IGA are likely to be pertinent mainly to Mediterranean basins. Yet, the main purpose of the study is testing the applicability of the IGA to basin management, and hence from a methodological perspective it is pertinent to other basin types as well.

In the next section we outline the IGA approach and the logic for identifying core issues as a basis for basin management. This paper focuses on the Hadera basin in Israel’s coastal plain. The third section thus opens with a brief description of the basin and then details the application of the IGA approach. We identify the core issues identified in the Israeli part of the Hadera basin case in the next section. In the following section the relevance of the core issues, as identified in the Hadera basin, and the extent to which they are transferable to other basins is discussed. This leads to conclusions regarding the utility and requirements, as well as limitations, of using the IGA approach to identify core issues in the river basin context.

## The Issue Generating Approach: An Overview of the Application to Basin Management

The IGA differs from conventional planning practices and is specifically suited to regional-scale planning. Rather than planners preparing a draft plan to which stakeholders are asked to comment, the IGA seeks to involve stakeholders in the preparation of the plan. The IGA follows thus the principles of the communicative planning approach, according to which the essence of planning is to communicate between the various stakeholders and to reach broad agreements, rather than to produce a plan. To this end the IGA uses the expertise of a wide array of professionals to focus stakeholder deliberations on core issues (Feitelson 2011). The communicative approach emphasizes participatory planning based on the assumption that collective wisdom is superior to the insights of a smaller planning team alone. Therefore, expanding the circle of participants is necessary for a learning process and promoting agreements regarding the goals and objectives, prior to the planning stage. The IGA, which comes to focus the participatory deliberations, has been implemented in the field of regional planning (Feitelson 2011) but does not appear to have been used in basin management.

IGA uses the process of evaluating alternatives in order to systematically and qualitatively identify the core issues that should be discussed in basin planning and management. The communicative planning approach requires evaluation of alternatives in a learning process in an arena that encourages constructive dialogue and open discussion. Rather than eliciting preferred options, the planning team provides the background knowledge and methodical framework for an educational experience that serves as an agreed-upon basis for planning.

The identification of core issues is necessary because spatial planning cases are multidimensional; there is no single optimal solution or outcome. The same is true for basin management, which is closely intertwined with land use planning (Padilha et al. 2023). Therefore, the traditional methods for evaluating alternatives, which focus on the choice of a preferred option ignore the reality of numerous potential response or solutions. With IGA, the evaluation is used to synthesize alternatives (Feitelson 2011). This synthesis is based on the systematic identification of the core issues in the basin and allows a response to be generated. Core issues are the most basic issues in the basin, the source of potential dilemmas and conflicts that have or may occur due to the various interests and needs of different stakeholders. Discussion of the core issues aims to reach broad understandings without directly delving into each local conflict. Focusing on core issues generates a sufficiently broad common denominator as a basis for planning, and thus potentially reduces contradictions between the various actors. Consequently, these understandings increase the likelihood of an agreed upon plan and the probability that the plan will indeed be implemented.

Core issues are identified in a process led by experts. Experts are those that are involved in basin management over time and thus are knowledgeable with regard to the basin. These can come from government, academia, basin management agencies, local government, non-governmental organizations (NGOs) or be water users. The experts make use of multi-criteria decision analysis (MCDA). However, instead of presenting a set of structured alternatives to choose from, they are presented with several extreme alternatives, intended to frame, and define the limits of the discussion. All stakeholders are asked to rank these alternatives quantitatively according to a series of criteria, and to justify their assessments. Unlike the conventional application of MCDA methods, in which the analysis is carried out quantitatively—summing the scores according to weights decided by the planning team (Aruldoss et al. 2013)—the IGA analysis is qualitative. The research procedure used as the basis for this article incorporated both quantitative and qualitative analyses, based on the assumption that reliance on a qualitative explanation for a quantitative rating would be more intuitive for respondents. Furthermore, this quantitative rating assisted the research team in more clearly understanding the respondent’s expressed position.

**Fig. 1:**
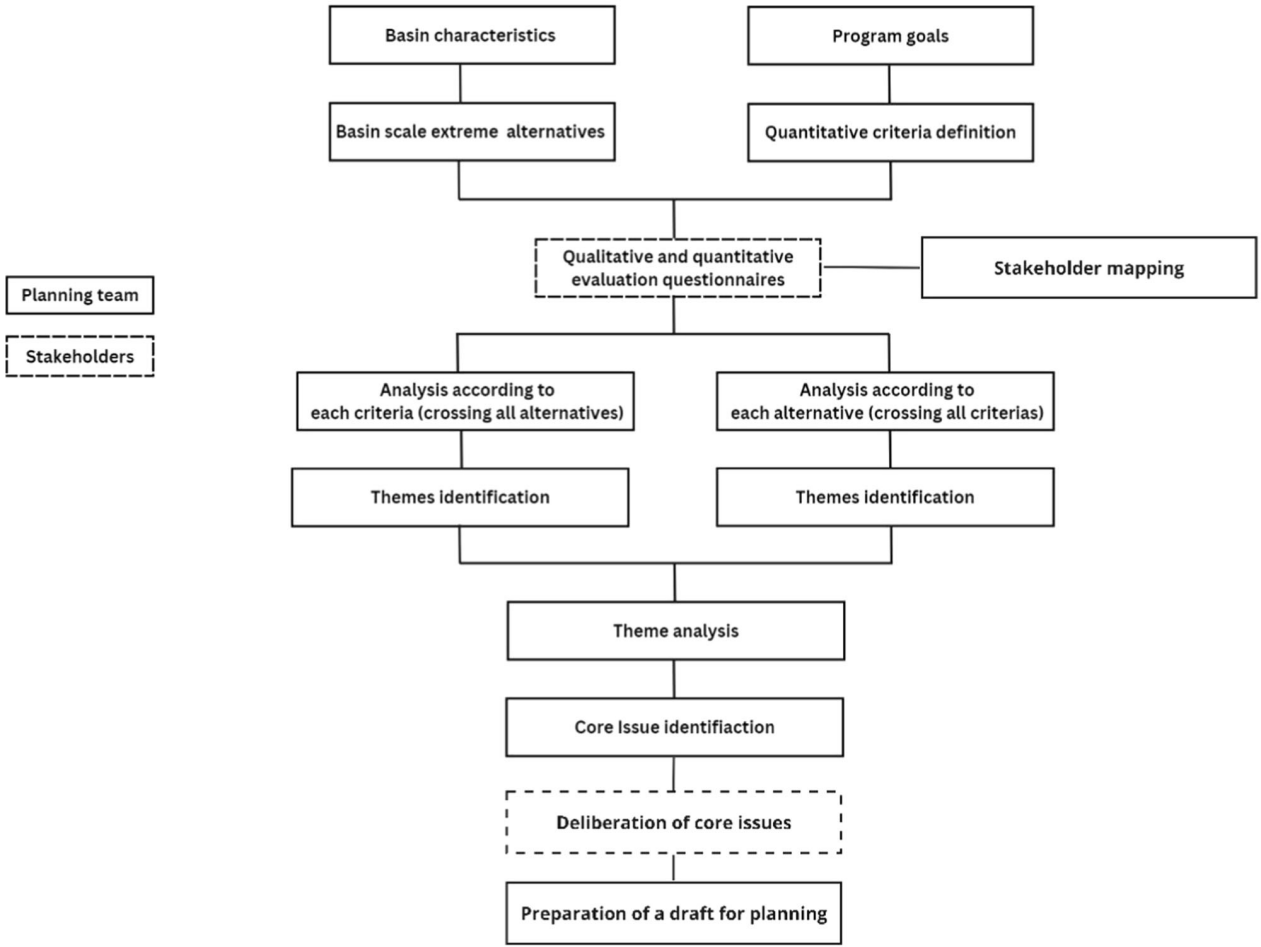
The core issue identification process according to the IGA approach

Using this method, a team of experts (the researchers in our case) analyzes the verbal justifications in a criss-cross method. First, all the comments regarding each criterion (across all alternatives) are summarized. Second, the comments regarding each alternative (across all criteria) are analyzed. The analysis seeks to identify attitudes and values which are represented in the comments expressed by the stakeholders and to point out the main themes, and particularly to identify the issues that underlie disagreements between the experts and potentially also between the stakeholders. These are the core issues. Based on an additional qualitative analysis of all the themes and issues identified, it is possible to reduce and refine the core issues and thus help focus further discussions among the stakeholders in an informed and evidenced-based manner. The stages of the application of the IGA approach are summarized in Fig. 1.

The core issues are intended to serve as the agenda for stakeholder workshops where they will be deliberated. In these deliberations (the broken bottom rectangle in Fig. 1) the stakeholders will try to reach agreements regarding the parameters that would then serve as a basis for the basin management plan. If these parameters are agreed upon it can be expected that the stakeholders will see the resulting plan as a plan they had a meaningful input, and thus will consent with the legitimacy of the planning process.

## The Hadera Basin case

**Fig. 2:**
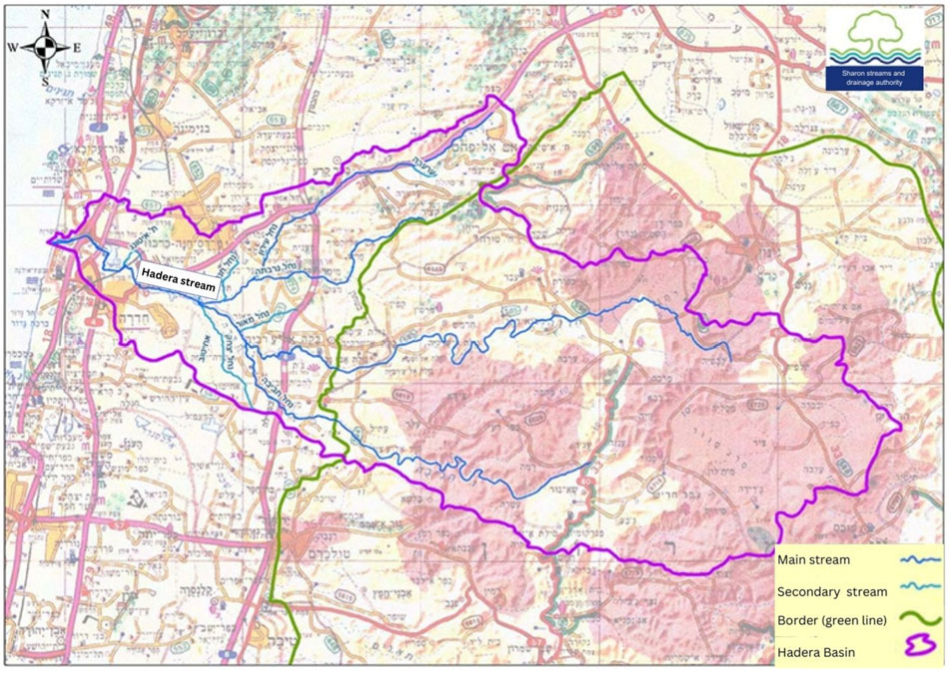
The Hadera basin area. This study focuses on the lower part pf the basin, west of the green line (Source: Sharon streams and drainage authority)

The Hadera basin is a transboundary basin, encompassing an area of about 600 km^2^ on both sides of the Green Line, the armistice line that separates the West Bank from Israel (Fig. 2) (Koller 2011). However, this study was conducted only in the western part (within Israel, west of the Green Line), which is the perennial part of the 60 Km long Hadera Stream that drains the basin (the eastern part being largely ephemeral). Moreover, this part is subject to a single planning system and under the jurisdiction of a single river and drainage authority.^1^ This part of the basin is densely populated containing both rural and urban Jewish and Arab settlements with an overall population of about 320,000 people.

In the past, the downstream channel of the river was characterized by wetlands and large seasonal bodies of water in an area of over 4000 dunams (4 sq km) with a diverse aquatic ecosystem (Koller 2011). Since the onset of the Zionist settlement of the lower part of the basin in the late nineteenth century, the wetlands (then viewed as swamps), were gradually drained, as water, particularly groundwater was increasingly utilized for irrigation, thereby lowering groundwater levels and reducing stream flows (Feitelson et al. 2014). In the 1950s the settlements in the area expanded and industrial plants were established. Municipal sewage and industrial wastewater were discharged into the various channels of the river. At the beginning of 1961, the Hadera River Drainage Authority was established, which channeled the lower part of the stream. In the 1960s to the 1970s the condition of the river continued to degrade as population grew and industries were established in the lower basin, resulting in an increase in the quantities of municipal and industrial wastewater, and fishponds expanded leading to higher water demand and increased groundwater extraction (Elron et al. 2015). Consequently, the stream was increasingly polluted and freshwater discharge fell substantially due to groundwater extraction, diversion of water for irrigation and aquaculture and floodwater captures upstream. Actions to reduce pollutants began in the mid-1980s with the allocation of financial resources for the restoration of coastal streams in Israel (Chenoweth 2006) the enactment of a new wastewater management regime (Hophmayer-Tokich 2010), with widened legal and spatial extent (Laster and Livney 2013). In recent years, sewage treatment facilities have been built and there has been an improvement in the water quality.

According to the water plan for Hadera stream (Elron et al. 2015), which pertains to the basin west of the Green Line, under the purview of the Sharon Stream Authority, the vision for restoration is to maintain a year-round base flow in the main tributaries without dams to divert the flow to agricultural plants and without pollutant discharges into the stream. Yet, even the anthropocentric goals for remediation, typical for Mediterranean coastal streams in Israel (Chenoweth 2006), have not been fulfilled as discharges from wastewater facilities and industry continue, as well as polluted return flows from agriculture. Moreover, with the increasing urbanization of the basin, coupled with expected increased extreme events due to climate change, flooding events and threats have increased (Elron et al. 2015). To address the expected increase in flooding a plan was recently prepared that focuses on retention in detention of floodwater.

## Applying the IGA: Methodology for Core Issue Identification

The identification of core issues in the Hadera basin case was undertaken in four steps: stakeholder mapping, development of the questionnaire (including identification of generic alternatives and criteria for evaluating the alternatives), stakeholder workshops, and analyses.

### Stakeholder Mapping

**Fig. 3:**
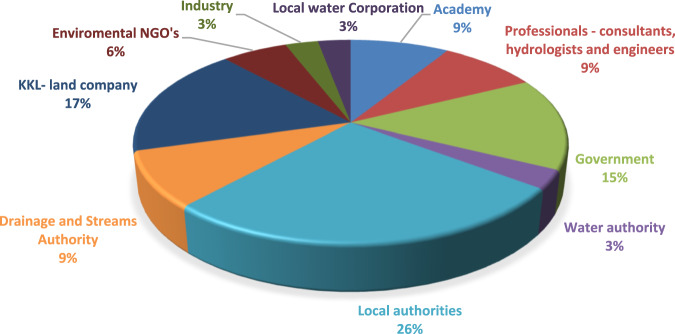
The stakeholders that participated in the study by organization (*N* = 34)

In the initial phase, the research team identified the relevant stakeholders in the Hadera basin through consultations throughout the basin with members of the river and drainage authority, NGOs and local authorities which took the form of snowballing, as well as the team members’ personal experience in the basin. Experts with different professional backgrounds (mainly planners, hydrologists, foresters, engineers) were invited from the various stakeholders identified, as well as experts involved in the basin due to their professional capacities and experience within the basin. A special effort was made to enlist experts from the Arab communities, all of which were contacted. Ultimately 67 main stakeholders were identified. Of these, 34 experts representing 14 different entities actively participated in the workshops^2^. The entities include the Shomron Drainage and Rivers Authority (which includes the Hadera basin), local authorities, the governmental water company Mekorot, water and sewer corporations, the Jewish National Fund (JNF), which is in charge of forestry, as well as various agricultural and civil organizations. The percent of participants by organization are presented in Fig. 3. As can be seen in Fig. 3 the largest number of participants came from local authorities.

### The Generic Alternatives and the Questionnaire

In the second stage the research tools were developed. The most important of these is the questionnaire, which served as a basis for the stakeholder workshops. The questionnaire presented three generic (extreme) alternatives, which were then assessed according to a set of criteria, which were also specified at this stage.

**Fig. 4:**
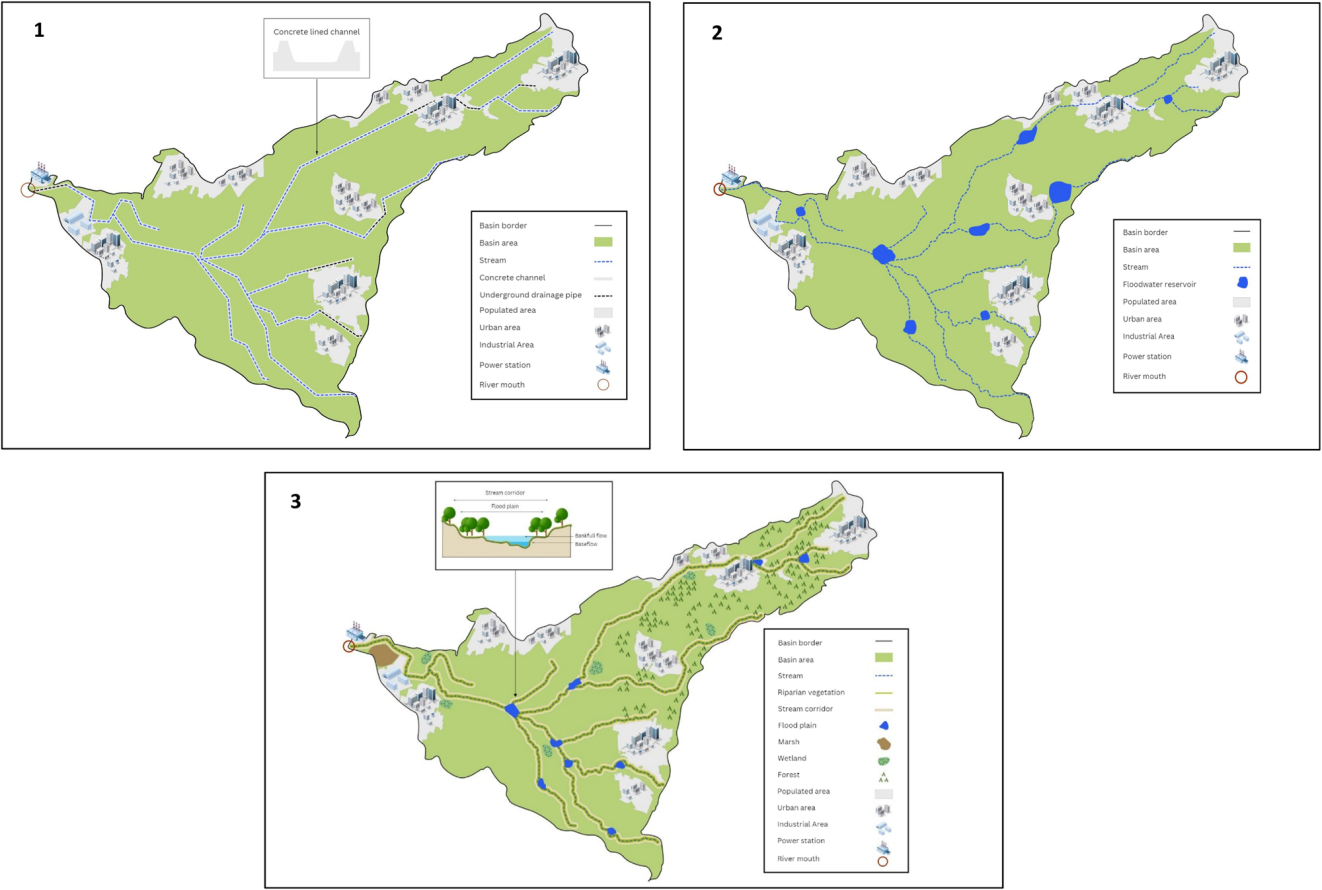
Schematic maps of the three extreme alternatives on a basin flow. A Hadera basin map was used as a base line, but municipal borders and identification of settlements, Agricultural plots and industrial properties were omitted to minimize objections. (1) Traditional drainage alternative includes channeling the stream channel with concrete and barring it in underground drainage pipe in urban areas to reduce flooding and maximize land use; (2) Floodwater reservoirs alternative includes engineering measures for detention and retention of floodwater for managing the water discharge downstream and reducing flood risks. They can be used for multipurpose such as water reuse and/or leisure parks and/or birds water source; (3) NBS for flood management includes a variety of elements to restore or mimic natural hydrological processes for flood risk reduction and the beneficiary of the natural ecosystem

The three generic alternatives represent three divergent approaches to basin management widely discussed in the literature (Wang et al. 2022): (1) traditional drainage using grey infrastructure for flood management, (2) floodwater detention and retention in multipurpose reservoirs, (3) the application of nature-based solutions (NBS) for flood management. The attributes of each alternative are presented in Table 1. Each alternative was graphically depicted, representing its implementation on a basin-wide scale (Fig. 4). These schematic maps of the Hadera basin provide an overview of the main features of each approach, as detained in Table 1. To enable a macro-level discussion and prevent place-based conflicts, no specific details, such as municipal borders or names of settlements, were included.

**Table 1:** The generic alternatives

Alternative	Main features
Traditional drainage approaches using grey infrastructure	Prioritizes flood risk reduction through rapid runoff drainage toward the sea using engineered solutions such as dams, stream channeling, and diversion. It aims to stabilize the stream geomorphology to establish a certain water discharge carrying capacity and to increase the development area by minimizing the floodplains of streams. It provides certain certainty through urban and rural protection using grey infrastructure.
Floodwater detention and retention reservoirs for multipurpose uses	Focuses on mitigating downstream flood risks by diverting the flow into upstream detention and retention reservoirs, thereby reducing peak flows. It emphasizes the multipurpose use of the reservoirs and the option of reusing reservoir water. It prioritizes the utilization of low-value land for reservoir location.
Application of nature-based solutions for flood management	Employs techniques in alignment with natural hydrological and morphological processes, features, and characteristics to manage flood water sources and pathways. It includes reducing runoff and delaying peak flows by preserving and restoring natural processes, conducting geomorphological and connectivity restoration of the channel, preserving natural floodplains, and undertaking wetland habitat restoration.

Next, the evaluation criteria were identified. These criteria were partially derived from the regulations governing stream and drainage management in Israel^3^. These criteria are detailed in Table 2, along with explanations for the way they were specified in the questionnaire. The explanations respondents provided for their assessment of the alternatives according to these criteria serve as a basis for the identification of core issues in basin management in settings such as found in the Hadera basin, that is Mediterranean settings.

**Table 2:** The criteria selected for the analysis of the three alternatives and the questions formulated for the stakeholders

Criterion	Questionnaire questions
Flood risk reduction	To what extent does the alternative reduce the risk of flooding in developed areas and provides protection for human life and property?
Development restriction	To what extent does the alternative allow construction?
Contribution to the water system by introducing runoff water into the aquifer and/or supplying water to agriculture	To what extent does the alternative contribute to the water system by introducing runoff water into the aquifer and/or supplying water to agriculture
Reduction of soil erosion	To what extent does the alternative contribute to reducing soil erosion?
Interface with agricultural activity	To what extent does the alternative enable and encourage agricultural activity?
Recreation activities by the river	To what extent does the alternative enable and encourage tourism and recreation in the area of the river?
Ecosystem and habitats protection	To what extent does the alternative preserve the natural ecosystems in the stream and along its banks?
Preservation and restoration of the ecological corridors	To what extent does the alternative help preserve and restore the ecological corridors?
Public health, removal of hazards and improvement of water quality	To what extent does the alternative contribute to public health by removing hazards and improving water quality?
Applicability	To what extent is the alternative applicable?
Construction costs	To what extent does the alternative involve establishment costs?
Maintenance costs	To what extent does the alternative involve maintenance costs (financial, manpower and knowledge)?
Public opinion support	To what extent will the alternative gain public support?
A comprehensive perception of the river basin	To what extent does the alternative encourage a comprehensive view of the river basin?

Prior to data collection, the questionnaire underwent a preliminary review by a panel consisting of representatives from the Drainage and Streams Authority, the Ministry of Environmental Protection, a professional independent hydrologist, and an independent city planner. This validation step ensured the questionnaire’s content and construct validity. In the full survey, the respondents were asked to rank each alternative according to every criterion on a scale, as is the practice in MCDM analyses and briefly explain their rankings. The MCDM results are presented in Table 3. As can be seen, the MCDM results favor a single, extreme, alternative (the nature-based solution). Yet, this outcome is of little practical value when there is a need to synthesize between the alternatives, as none of them is entirely applicable due to political and physical impediments (a point that was made in the workshops by the stakeholders during the open discussions).

**Table 3:** Results of the data analysis according to the MCDM method

Criterion	Traditional drainage approaches using grey infrastructure	Floodwater detention and retention reservoirs for multipurpose uses	Application of nature-based solutions for flood management	Variance
To what extent does the alternative reduce the risk of flooding in developed areas and provides protection for human life and property?	3.45	3.72	3.58	0.02
To what extent does the alternative allow construction?	3.84	2.97	1.90	0.94
To what extent does the alternative contribute to the water system by introducing runoff water into the aquifer and/or supplying water to agriculture	1.63	4.32	4.04	2.20
To what extent does the alternative contribute to reducing soil erosion?	2.34	3.16	4.03	0.71
To what extent does the alternative enable and encourage agricultural activity?	3.06	3.94	3.20	0.22
To what extent does the alternative enable and encourage tourism and recreation in the river?	1.97	3.44	4.61	1.76
To what extent does the alternative preserve the natural ecosystems in the stream and along its banks?	1.59	3.25	4.77	2.52
To what extent does the alternative help preserve and restore the ecological corridors?	1.78	3.48	4.63	2.06
To what extent does the alternative contribute to public health by removing hazards and improving water quality?	2.77	3.06	4.04	0.44
To what extent is the alternative applicable?	3.50	3.57	3.08	0.07
To what extent does the alternative involve establishment costs?	1.90	2.00	2.55	0.12
To what extent does the alternative involve maintenance costs (financial, manpower and knowledge)?	2.48	2.14	3.00	0.19
To what extent will the alternative gain public support?	2.50	3.45	3.83	0.47
To what extent does the alternative encourage a comprehensive view of the river basin?	1.94	3.38	4.50	1.65
Sum	34.75	45.88	51.77	

### Stakeholder Workshops

At the third stage, data collection was facilitated through stakeholder workshops rather than individual questionnaire distribution. The first of which served as a pilot for the main workshop. At each workshop, participants were introduced to the IGA research method’s guiding principles, in which the importance of qualitative explanations of the quantitative evaluation was emphasized. In addition the stakeholders could voice their perspectives in an open discussion. Thus the workshops served also as a communicative platform in line with the communicative planning approach which advocates for stakeholder engagement to facilitate shared understanding.

After a brief overview of the study’s background objectives and key IGA principles, the workshops were divided into four parts. In each of the first three parts, the focus was on one of the generic alternatives. In each of these three parts a detailed description of one of the alternative was presented, accompanied by a schematic map of its implementation within the basin (the relevant map in Fig. 4). Emphasis was placed on the alternative’s fundamental principles. Participants were then given time to complete the corresponding section of the questionnaire, either manually or digitally. This allowed for questions and clarifications while filling the questionnaire.

In the fourth part of the workshop an open discussion was conducted, which was systematically documented by the research team to supplement the analysis of the rationales that were analyzed on the basis of the questionnaires.

By leveraging stakeholder workshops as a data collection platform and effectively engaging participants in a meaningful dialogue, this approach provides a comprehensive and inclusive perspective on the issues at hand. The documentation of the open discussions further adds to the richness and comprehensiveness of the data collected, offering valuable insights into the stakeholders’ perspectives.

### IGA Data Analysis

The analysis of the qualitative explanations was conducted in two iterative rounds using a “criss cross” method. The method allowed a comprehensive analysis of the qualitative textual comments from the respondents according to each criterion (continuously transitioning between three alternatives), and subsequently of the insights gained from the qualitative text for each alternative.

In the first round, each stakeholder’s textual comments were thoroughly processed and categorized. All the comments were treated equally—regardless of the number of times the comment appeared. This process ensured the incorporation of a broad range of opinions and interests, regardless of the number of participants that hold any of the opinions. A list of comments was compiled, with keywords highlighting the identified standpoints. In the second round, each comment for every alternative was categorized based on its identified common theme, with separate categorizations for mentions of advantages and disadvantages. If a comment referred to two themes, it was categorized according to the main theme, except in cases where the secondary theme was unique.

**Fig. 5:**
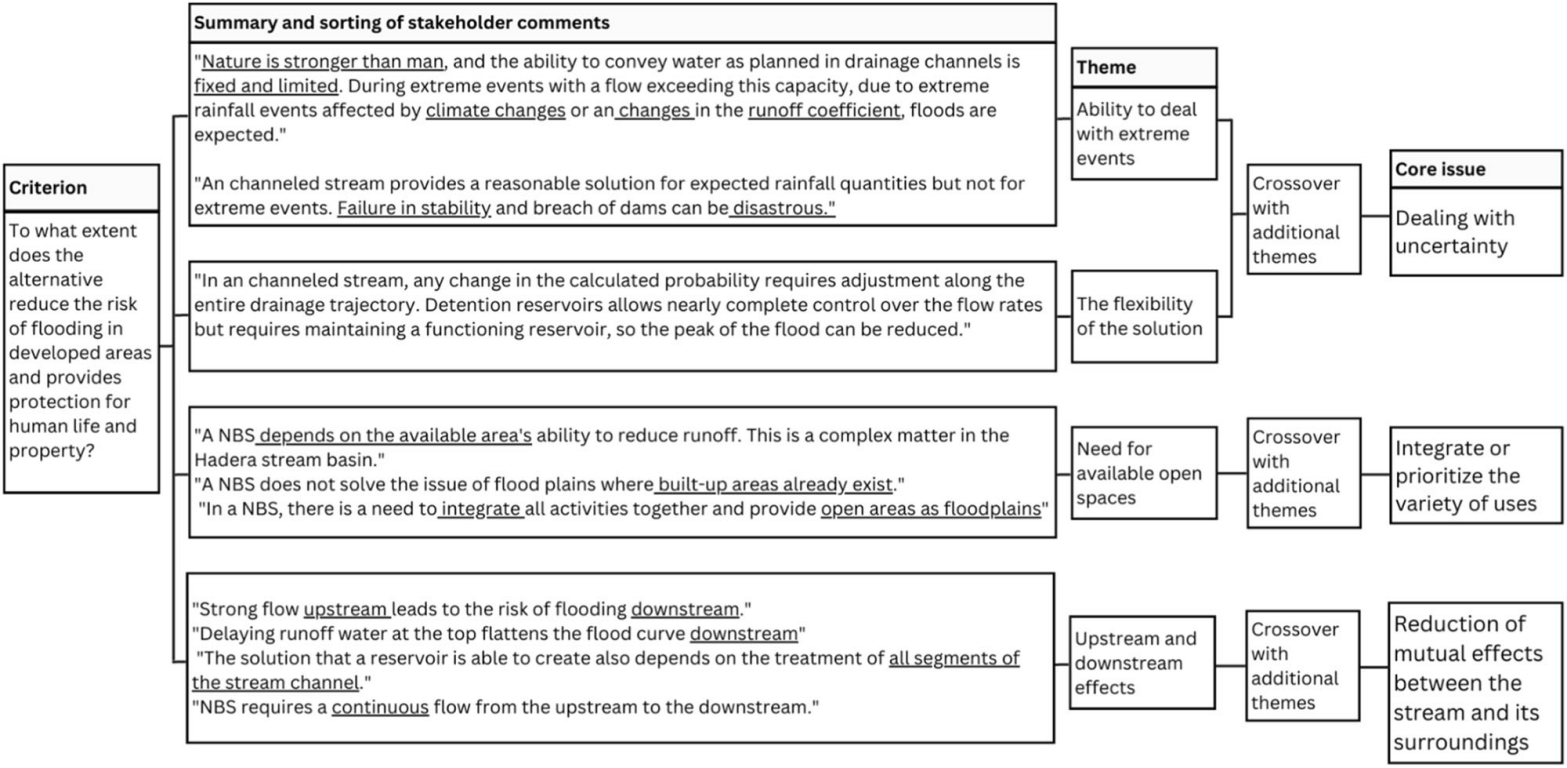
An example of a data analysis process according to a single criterion—flood risk reduction

Following this categorization, a list of 39 themes was created through cross-referencing the findings. This list was further sorted, and the 39 themes were divided into four categories based on the most basic and broad common denominator. This extensive categorization led to the identification of the core issues. For instance, the criteria of reducing flood damage to protect human life and property presented four distinct themes. When cross-referenced with additional themes, these were summarized into three core issues (Fig. 5).

To facilitate effective future basin-level discussions, a list of questions was formulated to present the dilemmas and complexities within each core issue, reflecting the uncertainties and disagreements regarding each issue. The issues and corresponding questions can serve thus as the basis for stakeholder dialogue. These are specified in detail in the next section.

## Results: The Core Issues

The outcome of the in-depth analyses and processing of stakeholders’ qualitative assessments, as described above, resulted in the identification of four central core issues:

(1) Managing uncertainty—addressing climate change and land use impacts on stream flow; (2) Mutual impacts management—handling interactions between the stream and its surroundings; (3) Integration of uses—balancing various stream utilization priorities; (4) Defining natural system functions—determining the role of natural systems.

To facilitate future deliberations with stakeholders, for each issue, a list of questions was formulated synthesizing and clarifying the content of the issue and the dilemmas arising from it. These questions are aimed at promoting discussion toward reaching agreements around a broad common denominator, as they should serve as the questions around which discussions should evolve in similar Mediterranean basins. In the remainder of this section the four core issues are specified, as well as the questions that have been put forward to discuss the each core issue.

### Managing Uncertainty—Addressing Climate Change and Land Use Impacts on Stream Flow

Both climate change and changes in land use, and particularly the expansion of impervious areas, are expected to alter stream flows (Ohana-Levi et al. 2018; Resaei et al. 2019). Both the extent and rate of changes in precipitation patterns in the Hadera basin as well as the rate and extent of building within the basin, and the impact such changes in land use will have on runoff coefficients and hence stream flow regimes, are highly uncertain. According to the stakeholders’ statements, grey approaches primarily involve engineering solutions for the fastest and most effective water drainage to the sea. These types of solutions are designed to address peak flows. Yet the probabilities of peak flows are based on past events and historic land use patterns. Therefore, under the assumption that the frequency of extreme events is expected to increase in the future as well as the extent of impervious areas, these solutions, which are limited in their flexibility, are likely to provide insufficient response in the long term. Other engineering interventions such as reservoir construction enhance the range of events that can be accommodated, thus helping to cope with uncertainty. But also in this case the storage capacity for which they are built is a function of past events and land use patterns, which will not accurately reflect future events and runoff coefficients, and thus some excess capacity should be planned. Yet what this excess capacity should be is uncertain. Nature-based solutions were perceived by stakeholders as sustainable, but compared to engineering solutions, which are well-known for which much local experience has accumulated; uncertainty arose regarding the control capacity of these solutions, the extent to which they mitigate the rise of runoff coefficients, their economic costs, and the land resources they require.

In order to advance a systematic and focused discussion with stakeholders on this issue, a list of questions was formulated that can assist in reaching local-level agreements, while taking a broad perspective on the topic. These are detailed in Box 1.

By addressing these questions local stakeholders may be able to reach an understanding regarding the parameters that will guide local plans, such as the extent to which safety margins should be extended. That is, if current safety margins take a once to 50 years flood as a benchmark, for example, as such floods may become more frequent another benchmark may be more suitable in future plans, and particularly infrastructure projects. The decision on which flood frequency to base such margins under the current uncertainty is a decision that needs to be agreed upon as a basis for planning. By addressing this question an early agreement among stakeholders may reduce disagreements down the road.

Box 1: Guiding Questions for Discussion of Climatic and Land Use Uncertainty

- Which areas are most susceptible to damage during extreme events?
- To what extent are different areas resilient to damage during extreme events?
- Is there a need to increase the safety margin in order to reduce the risk of flooding in sensitive areas? By what means? What are the implications of such additional safety margins?
- What methods of drainage and stream management allow for the required actions and economic flexibility? Are there knowledge gaps on this topic?
- What is the appropriate and feasible strategy for dealing with sensitive areas in flood-prone regions?
- What are the appropriate and feasible means of managing upstream drainage in a way that does not exacerbate downstream flows?

### Mutual Impacts Management—Handling Interactions Between the Stream and Its Surroundings

It is evident from the stakeholders’ statements that they agree that the stream and its surroundings influence each other; the stream is primarily affected by soil erosion, the amount of discharge, and pollutants discharged into the channel by activities upstream. The stream’s surroundings may be subjected to flooding or affected by hazards that can occur around the stream, for example, due to standing water (Pargament et al. 2010). In order to mitigate these undesirable interactions there is a need to undertake actions that will reduce these interactions in the interface between the streams and their surroundings.

The dilemmas regarding this issue revolve around identifying areas where intervention, such as buffer zones, are needed, the methods required to identify and establish such interventions (buffers), and how to cope with the consequences of these interventions. In essence, this interface requires that actions will either be undertaken along the streams, such as buffers or upstream, at the source.

Yet, the ability to undertake such actions is a function of the structures of property rights, as well as of the specifics of the actions taken, such as types of vegetation along the streams and the specific goals that such buffers are set for (Mundahl et al. 2023). That is, to what extent can and should the regulator intervene with regard to buffers and agricultural practices, who should the regulator be, and what should the intervention approach be? Should a regulative approach be undertaken, and if so under which statute or should an incentive or tradeable property rights approach be taken? The answers to such questions are clearly normative, and hence they should be addressed as a basis for the management regime that is put in place.

To construct a systematic discussion with stakeholders on this issue, a list of questions raising the dilemmas noted above was formulated, intended to promote the deliberations among stakeholders. These are specified in Box 2.

Box 2: Guiding Questions Regarding the Interactions Between Streams and Their Surroundings

- To which issues can a response be provided at the source? How?
- Where are buffer strips required? What is the necessary width of the buffer?
- What are the implications of establishing buffer? How can we deal with them?
- The establishment mechanism in private and state lands: (a) How and in which cases will a compensation mechanism be applied when lands in private ownership are expropriated? (b) How and in which cases will a compensation mechanism be applied as a result of flooding damages to agricultural lands owned by the state? (c) To what extent does the political and social reality allow the application of these mechanisms? (d) Can a different mechanism be implemented to reduce conflicts?
- To whom will the authority and responsibility for managing the area be given?
- What funding, management, and maintenance mechanism can be activated in the buffer zone?

### Integration of Uses—Balancing Various Stream Utilization Priorities

The integrated basin management approach that incorporates all uses of the river basin is an approach that encompasses the entire spectrum of usage within the basin, based on an understanding of the relationships and mutual influences among a variety of uses in the basin (Heathcote 2009). Yet, there are conflicts between the various activities within the basin. Consequently, there is a need to address these conflicts in order to advance integrated management of the basin, as well as to identify possible synergies which can be realized in a basin management plan (Jaspers 2003). From the stakeholders’ perspectives, there is a need to address these conflicts in the planning stage by prioritizing or identifying multiple beneficial interfaces and synergies, as well as the conflicts between activities and actions (Padilha et al. 2023). An example that was repeatedly mentioned by stakeholders is the complex interface between tourism and recreation and agricultural areas (productive ecosystem services), or between tourism development and the conservation of sensitive ecological systems, and particularly aquatic ecosystems.

To discuss this issue within a communicative framework, several guiding questions were defined. These questions, detailed in Box 3, can help to structure the discussion in defining target audiences, required needs and responses, along with management implications. Addressing these questions is intended to help identify points of connection between different uses, or alternatively, to prioritize uses according to different segments in the basin, with a broad basin-wide perspective.

Box 3: Guiding Questions for Discussing Stream Utilization Priorities

- Who is the main target audience that the specific part of the basin is expected to serve? What uses should be prioritized in this part of the basin?
- Is there a high cultural value or public interest in the basin section being discussed that should be expressed?
- In which areas is there an incentive or interest in developing tourism? Is it relevant all year round?
- What type of tourism be designed?
- What mechanism can be implemented to finance the operation and maintenance of areas designated for tourist activity or recreation and leisure?
- Are the areas where there tourism development is likely to harm other uses? How can the negative impacts be minimized?
- Can the water reservoirs be used for additional purposes? (For example—water reuse, a bird sanctuary, a scenic attraction point, boating)?
- Where and how can uses be intersected to promote multiple benefit spaces?
- Are there entities with similar interests that can connect to multiple benefit activities in the basin and pool budgets?

### Defining Natural System Functions—Determining the Role of Natural Systems

Through the various stakeholder engagements, we observed that terms like “nature”, “natural”, and “healthy river” were predominantly viewed in a positive light. These terms emerged in the context of enhancing attractive recreational spaces, improving ecosystem services, bolstering resilience against climate change, and addressing the needs of aquatic ecological systems. However, the analysis of stakeholder statements also revealed a lack of uniformity in defining what a “natural” occurrence is and entails. Some stakeholders referred to nature with minimal or no human interference, while others proposed semi-organized tourist sites or well-managed locations, which are essentially created and managed by humans.

The differences in the way nature is perceived have wide implications for the practical management of the basin. The implications range from the landscape that is fostered (wild nature, landscaped nature, parks) through the intended beneficiaries (wildlife, hikers, families) to the practicalities of management. That is, who should finance and how should various landscapes be financed (through market-based mechanisms or from the general revenue, and through which agency), who should be responsible for maintaining the streams and their environs, and should the streams be viewed as a unitary resource or should different parts of the basin be treated differently, and if so on what basis.

The discourse revealed that several guiding questions could help structure further deliberations on this matter. The importance of these questions lies in two main domains. Firstly, they assist in creating a unified and common language for all stakeholders, fostering enhanced understanding and collaboration. Secondly, they aid in aligning expectations regarding the desirable natural occurrences, necessary ecosystem services, and resource allocation planning intended for this purpose. The questions that can structure deliberations on these issues are detailed in Box 4.

Box 4: Guiding Questions for Discussion on the Definition of Natural in Basins

- What type of natural landscape is the goal of the project (wild nature, managed nature, nature accessible to visitors, parks, etc.)?
- Who is the intended beneficiary of the restoration efforts?
- What are the principal ecosystem services these areas are intended to provide?
- What kind of habitats are desirable to preserve or restore? Can we define core areas for restoration? How should we plan the space to allow this?
- Is it desirable and feasible to maintain or prioritize a sequence of open spaces in the basin to allow connectivity?
- What are the viable natural restoration objectives within the project framework (timeline, budget, constraints)?

## Discussion and Conclusions

Stakeholder engagement is a central tenant in basin management (Jonch-Clausen and Fugl 2001; Lim et al. 2022). Much discussion has focused on the representation of stakeholders or lack thereof and the need to improve representation (Blackstock et al. 2014; Schroder et al. 2020). Nevertheless, it is insufficient to merely bring the various stakeholders to the table. Rather, for a meaningful outcome from stakeholder engagement stakeholder meetings should be well structured in order to facilitate the management of the basin and to find common understandings, rather than amplify conflicts (Wehn et al. 2015). By implementing the IGA method we identify core issues that should be discussed by stakeholders in basin management. Clearly, there is no “right” of “wrong” answer to any of the core issues. Rather, they are dilemmas that stakeholders need to deliberate and if possible reach agreement on regarding the responses for them. To facilitate these discussions guiding questions are proposed for each core issue. These questions, as well as the core issues, are not exhaustive. Moreover, the way they are addressed will differ by the specifics of the basin and the stakeholders involved. Yet, they may serve as a starting point for stakeholder deliberations in multiple settings. Moreover, by implementing the IGA method, the study assimilated collaboration and collective thinking from the outset, thereby enhancing the social and political capital of participants in line with the communicative planning approach (Healey 1992).

Unlike traditional data analysis methods that often require a large number of respondents, the IGA method prioritizes the representation of interests and disciplines to elicit ideas over participant quantity (Feitelson 2011). Accordingly, considerable effort should be made to ensure comprehensive representation across various interests and from different vantage points, strengthening the validity of the findings. In this study, the core issue identification focused specifically on stream management, a crucial component of integrated basin approaches. By developing three generic alternatives and subjecting them to cross-examination against 14 criteria, stakeholders’ perspectives are assessed, encouraging a focused and constructive discussion. In our case 34 experts participated, out of the 67 invited.^4^ Clearly participation can be enhanced by conducting additional workshops. However, in a previous application of the IGA reported by Feitelson (2011) the addition of participants was found to have diminishing returns as participants in later workshops increasingly reiterated statements made earlier. As the purpose of the IGA is to set the agenda for further deliberations, those where a wider representation will be sought, and a wide array of statements was obtained, the need for additional workshops was not seen as critical. This contrast with the need for representation at later stages (the bottom box in Fig. 1), the stage in which the core issues identified through the IGA are deliberated.

The core issues identified in this study stem from the specific characteristics of the Hadera stream basin, and thus may be pertinent also for other Mediterranean coastal basins. When examining the identified issues, it becomes evident that they contain elements of coping with uncertainty alongside concerns about the exploitation of limited resources. These topics are particularly relevant in Mediterranean basins characterized by substantial seasonal and annual precipitation and temperature variations, which directly influence flow dynamics in streams (Cid et al. 2017; Bonda and Resh 2103; Kondolf et al. 2013) the fluctuations of which are likely to be accentuated due to climate change (Döll and Schmied 2012). Yet, these characteristics play a central role in shaping ecosystems and human communities in most climatic regions globally (Poff et al. 1989). Thus, the core issues identified in our study may be relevant also in other settings. However, the extent to which they are indeed valid in other climatic regions requires additional applications of the IGA in such regions.

To address the complexity of each core issue we advance a set of questions to facilitate a comprehensive discussion of the issue, without digressing to the very local conflicts that often arise in stakeholder meetings. Implementing this approach during early planning stages may expedite the process by reducing potential opposition compared to pre-selecting detailed alternatives as the early deliberations around the dilemmas raised by the core issues can form an agreed-upon basis for the basin management plan. Additionally, this method fosters effective communication of stakeholders’ perspectives, aligning them with local interests and guiding the formulation of principles for ongoing planning and basin management. The method thus minimizes biases that might arise from quantitative evaluations of alternatives (Feitelson 2011). Furthermore, it curtails the influence of the specific planning team or facilitator, promoting an impartial and inclusive discussion.

Despite the strengths of the IGA method, certain limitations should be recognized. The dialogue’s scope is constrained by predefined criteria and the specification of the proposed generic alternatives, potentially overlooking relevant basin issues. To mitigate this concern, a preliminary basin assessment was conducted to identify any overlooked topics and ensure a more inclusive representation of stakeholder interests. As the purpose of this study was to examine the feasibility and applicability of the IGA to basin management it was not part of comprehensive plan. The next stage will thus be to utilize the results of an IGA as a basis for actually planning the basin, which will allow for a better assessment of its contribution.

Looking ahead, the insights from this research have significant implications for future studies and applications in similar basins. A more comprehensive and meaningful communicative process could be achieved by initiating the dialogue from the outset, facilitating a finer delineation of generic alternatives and relevant criteria, ultimately leading to a more robust and exhaustive debate. Furthermore, conducting a comparative analysis of findings from similar would be instrumental in developing a broader and richer list of core issues and guiding questions.

Participatory processes, widely viewed as central in the integrated basin management and integrated water management literature has been found to be often highly fragmented, and with limited results on decision making (Lim et al. 2022; Schroder and Watson 2024). In this research we advance a method, the IGA, whose intention is to identify the core issues that demand attention in basin management at the outset of the planning process. It structures thus stakeholder discussions in a manner that potentially fosters meaningful and productive deliberations to reach common understandings over issues at the heart of basin management as a basis for basin planning. By structuring stakeholder participation around the core issues participants become partners in the planning process, thereby moving such participation up on Arnstein’s (1969) ladder. Still, to the extent that the participants in the IGA process are not sufficiently representative of the interests and expertise that should be involved in the management of the basin the IGA may further power imbalances by leaving out core issues that may be relevant and will thus not be discussed. While the core issues identified in this study are likely to be pertinent mainly to Mediterranean streams the approach through which they were elicited (the IGA method) can demonstrably be used in other climatic zones as well. One lacuna that should be addressed in future studies is the application of the IGA approach to transboundary settings. Through this approach, we offer valuable insights into stakeholder engagement and core issue identification, thereby contributing to the advancement of meaningful stakeholder participation in integrated basin management.

## Data Availability

No datasets were generated or analyzed during the current study.

## Footnotes

1 The eastern part of the basin is within the West Bank, and thus is controlled in part by the Israeli Civil Administration and in part by the Palestinian Authority. There is no operational basin authority in this part of the basin.

2 Two workshops were held, the first one as a pilot. As the stakeholders contacted are mostly busy professionals or elected officials scheduling proved to be a challenge. Due to the small number of Arab participants professionals in the Arab jurisdictions were contacted directly beyond the workshops. However, these efforts were only partially successful, as only four Arab professionals ultimately evaluated the alternatives.

3 These regulations included The Drainage and Flood Protection Law (1957), Land Conservation Regulations (1960), and the Law of Rivers and Springs Authorities (1965-1965).

4 These experts came from the Israeli part of the basin, which is the perennial part of the stream and is managed by a single river authority. Thus the core issues pertain only to this part of the basin. Clearly core issues which will pertain also to the management of the Palestinian part of the basin are likely to be wider as they will pertain also to the transboundary nature of the basin. Yet, the incorporation of trans-boundary aspects are left to future studies.
